# Lowered social motivation is associated with adolescent attention deficit hyperactivity disorder and social anxiety symptoms

**DOI:** 10.1177/13591045231218475

**Published:** 2023-11-23

**Authors:** Rachael Martin, Erin McKay, Hannah Kirk

**Affiliations:** Turner Institute for Brain and Mental Health, School of Psychological Sciences, 2541 Monash University, Clayton, VIC, Australia

**Keywords:** ADHD, social motivation, social skills, attention, development, adolescence, motivation, intervention

## Abstract

**Background:**

Difficulties in social skills are highly prevalent in children and adolescents with attention deficit hyperactivity disorder (ADHD), however, the reasons for these social difficulties are poorly understood. This study aimed to understand social motivation in children (aged 5–8) and adolescents (aged 13–17) with and without ADHD, and the relationship between social anxiety and social motivation in youth with ADHD.

**Method:**

204 parents of children and adolescents with and without ADHD completed online questionnaires on social motivation, social anxiety, and ADHD symptoms.

**Results:**

Adolescents with ADHD had significantly lower social motivation than typically developing adolescents, and children with ADHD. Higher social anxiety significantly predicted lowered social motivation in participants with ADHD.

**Conclusions:**

These findings indicate a unique pattern of social motivation in ADHD, specifically a reduction during adolescence, reflecting social intervention inefficacy. Additionally, these findings establish a link between social motivation and social anxiety, suggesting that they may act as barriers to social intervention.

## Introduction

Developing age-appropriate social skills during childhood and adolescence is integral to academic performance, mental well-being, and overall quality of life (Tan et al., 2018). Difficulties establishing appropriate social skills during this critical period are associated with greater peer rejection (Vanhalst et al., 2015), school maladjustment (Rokeach & Wiener, 2022), increased risk of antisocial behaviours, and academic difficulties (Gardner & Gerdes, 2015). Recent research has therefore focused on understanding the social profiles of children and adolescents with social difficulties, such as those with attention deficit hyperactivity disorder (ADHD; Aduen et al., 2018).

ADHD is the most common childhood neurodevelopmental disorder, with an estimated global prevalence of 5.9% in children aged under 18 (Faraone et al., 2021). Although ADHD is characterised by pervasive difficulties in attention and/or hyperactivity/impulsivity (American Psychiatric Association, 2013), difficulties in social skills are a central feature and are noted as a significant concern by parents (Bora & Pantelis, 2016). As individuals with ADHD enter adulthood, social difficulties are associated with diminished emotional recognition and social reasoning (Thoma et al., 2020), leading to reduced performance in workplace settings (Fuermaier et al., 2021). Therefore, the consequences of social difficulties in ADHD are pervasive and persistent, indicating a need to understand and address potential underlying contributors to social difficulties.

The social difficulties experienced by individuals with ADHD have been attributed to a variety of underlying factors, such as deficits in social cognition (e.g., emotional perception and social knowledge; Bora & Pantelis, 2016), maladaptive ADHD symptomatology (e.g., excessive hyperactivity and impulsivity; McQuade & Hoza, 2015), and difficulties in social performance (i.e., failure to enact social knowledge; Aduen et al., 2018), however a recent study has implicated the role of social motivation (McKay et al., 2023). Social motivation is a core element of social functioning, and refers to the seeking, fostering, and enjoyment of social interactions (Chevallier et al., 2012). Social motivation is essential to developing appropriate social skills as it promotes enhanced attention to social stimuli (e.g., human faces and voices), the development of social information processing and cognition systems (e.g., facial processing and perspective taking), and motivates individuals to engage in peer interactions (Leary & Allen, 2011). Therefore, individuals experiencing lower levels of social motivation often exhibit greater social difficulties (Leary & Allen, 2011). To date, this association has not been explored in children and adolescents with ADHD, despite their well-documented social difficulties.

In typically developing individuals, social motivation follows an upward trajectory from birth, reaching a peak in adolescence and declining during early adulthood (Ciranka & van den Bos, 2019). During the peak of their social motivation, typically developing adolescents are motivated to foster relationships, appear socially desirable to their peers, and gain popularity; all of which bolster social skills (Leary & Allen, 2011; Vanhalst et al., 2015). However, some research suggests individuals with ADHD may experience altered trajectories of social motivation (Chevallier et al., 2012; Clements et al., 2018). Despite children with and without ADHD experiencing similar levels of social motivation (Smith & Langberg, 2018), adolescents with ADHD exhibit significantly lower levels of social motivation than their typically developing peers (McKay et al., 2023). As such, social motivation may decline rather than peak during adolescence for those with ADHD. However, no study to date has compared social motivation in ADHD across developmental periods, making it difficult to understand its age-related differences within this population (Morris et al., 2021; Storebø et al., 2019).

The presence of social anxiety symptoms may further perpetuate the low social motivation present in adolescents with ADHD. Social anxiety symptoms, which include pervasive fears surrounding peer appraisal, social embarrassment, and social performance (American Psychiatric Association, 2013), are often present in ADHD, with up to 50% of children and adolescents presenting with ADHD also meeting the criteria for social anxiety disorder (Mojahed et al., 2021). The relationship between social anxiety symptoms and social skills difficulties in typically developing youth is well-established; with social anxiety symptoms bi-directionally associated with increased avoidance behaviours (Greenberg & De Los Reyes, 2022) and poorer social skills (Klein et al., 2021). In individuals with ADHD, social anxiety symptoms are similarly associated with reduced social skills (Becker et al., 2015), social isolation (Lau et al., 2022), and diminished self-esteem (Çelebi & Ünal, 2021). Social anxiety therefore compounds the social difficulties experienced by children and adolescents with ADHD, however the relationship between social anxiety and social motivation in this population remains unclear, potentially impacting treatment response.

Should social motivation differ between children and adolescents with ADHD, and typically developing children and adolescents, this may impact treatment response. Currently, there is little evidence to support the efficacy of social interventions for individuals with ADHD, with recent literature highlighting that interventions are even less effective in adolescents with ADHD and social anxiety (Bishop et al., 2019; Mikami, 2015). It may be that lowered levels of social motivation reduce the likelihood of adolescents with ADHD to engage in and perform learnt skills outside of intervention sessions, thereby reducing their impact on improving social skills (Devine & Apperly, 2022). Accordingly, rather than focusing on developing new social interventions, researchers and clinicians may be able to improve social intervention efficacy in adolescents with ADHD by examining the barriers to social performance and intervention participation. Preliminary findings into the potential mechanisms underlying social intervention inefficacy in children and adolescents with ADHD and social anxiety symptoms suggest that the social avoidance behaviours associated with social anxiety (e.g., declining social invitations, remaining quiet in social conversations) may act as barriers to performing learnt skills outside of intervention sessions (Bianchi et al., 2020; Bishop et al., 2019; Mikami et al., 2017). However, it is unclear whether these avoidance behaviours are related to social motivation, and therefore whether a relationship exists between social motivation and social anxiety in children and adolescents with ADHD.

Given the high prevalence and negative consequences of social skills difficulties in children and adolescents with ADHD, understanding their contributing factors during this critical period is of great importance. Through comparing social motivation between children and adolescents with and without ADHD, the present study will advance knowledge and understanding of the social difficulties within this population, and highlight the possible age-related differences associated with social motivation in youth with ADHD. This holds implications for the implementation and design of social interventions, as if social anxiety symptoms are found to be associated with reduced social motivation in children and adolescents with ADHD, then social interventions may be bolstered by pairing them with interventions to reduce social anxiety. Additionally, previous research into the social skills of children and adolescents with ADHD has mainly focused on self-reports of social functioning, despite indications that children and adolescents with ADHD often overestimate their competencies in social domains (Du Rietz et al., 2016). The present study will therefore add novel insight to the existing literature through use of parent reports to measure social motivation, social anxiety, and social difficulties in children and adolescents with ADHD.

This study therefore aimed to firstly explore potential differences in social motivation between children and adolescents with and without ADHD, and secondly examine associations between social anxiety and social motivation in children and adolescents with ADHD. It was hypothesised that social motivation would be significantly lower in adolescents with ADHD (13–17 years) compared to both typically developing adolescents (13–17 years), and children with ADHD (5–8 years). It was also hypothesised that higher social anxiety symptoms would be associated with lower social motivation in children (5–8 years) and adolescents (13–17 years) with ADHD.

## Method

### Participants

Respondents in the current study were 204 parents of children and adolescents with and without ADHD residing in Australia; parents of children and adolescents with ADHD self-reported the ADHD diagnosis of their child. Respondents were excluded if their child (1) did not reside with them for the majority of the week (<3 days), (2) had a diagnosis of autism spectrum disorder, (3) had a history of major trauma, (4) had a diagnosed intellectual disability or global developmental delay, or (5) had a sibling completing the study. See Table 1 for participant demographics. This study was approved by the (BLINDED) Human Research Ethics committee.

**Table 1.** Participant Means, Standard Deviations, Frequencies, and Proportions for Demographic Characteristics Across Sample Groups.

	TD children (*n* = 43)	Children with ADHD (*n* = 49)	TD adolescents (*n* = 36)	Adolescents with ADHD (*n* = 76)
Age in years M(SD)	6.09 (1.02)	6.92 (.91)	14.81 (1.49)	14.42 (1.34)
Gender *N* (%)				
Male	23 (53%)	29 (59%)	18 (50%)	29 (38%)
Female	20 (47%)	20 (41%)	17 (47%)	43 (57%)
Gender diverse	0 (0%)	0 (0%)^a^	1 (3%)	4 (5%)
ADHD type *N* (%)				
Inattentive	-	4 (8%)	-	29 (38%)
Hyperactive/Impulsive	-	4 (8%)	-	8 (11%)
Combined	-	15 (31%)	-	34 (45%)
Unsure	-	26 (53%)	-	5 (7%)
Attentional difficulties *N* (%)	17 (40%)	49 (100%)	19 (53%)	76 (100%)
Social impairment severity *N* (%)				
Within normal limits	30 (70%)	20 (41%)	23 (64%)	14 (18%)
Mild	9 (21%)	11 (22%)	7 (19%)	15 (20%)
Moderate	4 (9%)	10 (20%)	4 (11%)	31 (41%)
Severe	0 (0%)	8 (16%)	2 (6%)	16 (21%)
Medication status *N* (%)^b^				
No	-	7 (14%)	-	13 (17%)
Yes	-	29 (59%)	-	63 (83%)

Note. *N* = 204; M: mean; SD: standard deviation; ADHD: attention deficit hyperactivity disorder; TD: typically developing; attentional difficulties were measured by the Developmental and Wellbeing Assessment ADHD Section; values under attentional difficulties indicate the proportion of the sample with attentional difficulties; social impairment was measured using the Social Responsiveness Scale – second Edition severity scores due to the presence of gender diverse participants in the sample.

^a^Only male and female gender options were provided to this sample, so gender diversity could not be measured.

^b^13 children with ADHD did not have data in relation to medication status.

### Materials

Screening and Demographics questionnaires were completed to determine study eligibility and the demographic characteristics of the sample.

Social Motivation was assessed using the parent report Social Responsiveness Scale, second Edition (SRS-2; Constantino & Gruber, 2012). The SRS-2 is designed for use in children aged 4–18 years, and comprises 65 items across the five subscales of social behaviours (Social Awareness, Social Motivation, Social Cognition, Social Communication, and Restricted Interests and Repetitive Behaviours). Parents rate their child’s social behaviour over the last 6 months using a 4-point Likert scale (‘*not true*’ – ‘*almost always true*’ [0–3]). Raw scores from the Social Motivation subscale were used in analysis, whereby higher scores indicate greater impairment (i.e., lower social motivation). Severity ratings of social difficulty for group comparisons and sample characterisation (see Table 1) were determined using the total score descriptors, derived from the total T-score, as these are appropriate for use in gender diverse samples. The SRS-2 has high concurrent validity and strong internal consistency (α = .94 to .96) in child and adolescent populations (Bruni, 2014).

Social Anxiety Symptoms were measured using the social phobia subscale of the Spence Children’s Anxiety scale, parent-report (SCAS-P; Nauta et al., 2004; Spence, 1998). The Social Phobia subscale comprises six items on a 4-point Likert scale (‘*never*’ – ‘*always*’ [0–3]), where higher scores indicate greater social anxiety symptoms. Raw scores were used in analysis. The SCAS-P has high convergent and divergent validity, and acceptable internal consistency in children and adolescents (α = .74; Nauta et al., 2004). Parent report of anxiety symptoms has been found to be a significant predictor of anxiety disorder diagnosis (Hyland et al., 2022), and are a valid approach measure of anxiety symptoms in children and adolescents (Hyland et al., 2022; Weems et al., 2010).

ADHD Symptoms were measured using the ADHD section of the Developmental and Wellbeing Assessment (DAWBA; Goodman et al., 2000); a structured parent interview available for online use as a questionnaire in children aged 5–17. The DAWBA has high predictive and concurrent validity, and acceptable reliability in the current population (Aebi et al., 2012; Goodman et al., 2000).

### Procedure

Respondents accessed the link to the explanatory statement and aforementioned online questionnaires via the study advertisement which was posted online to social media groups. Consent was implied through the completion and submission of the questionnaires rather than a consent form to maintain the anonymity of the participants. Questionnaires were administered online via REDCap (Research Electronic Data Capture), a secure, web-based application for research studies hosted and managed by Helix (BLINDED).

### Data analysis

Statistical analyses were performed using R Studio v4.2.0 and IBM SPSS Statistics v28. Statistical significance was set at α = .05 for all analyses. Demographic characteristics of the sample were assessed using descriptive statistics, and chi-square tests and t-tests were run to determine the presence of any significant differences in demographic characteristics across participant groups (Field, 2009). Cramer’s V was used as an effect size for chi-square comparisons (i.e., gender, ADHD type, attentional difficulties, social difficulties, medication status), and Cohen’s *d* was used as an effect size for *t* test comparisons (i.e., age).

To investigate social motivation differences between children and adolescents with and without ADHD, a one-way ANOVA was conducted, comparing differences in raw SRS-2 Social Motivation subscale scores between children with ADHD, adolescents with ADHD, typically developing control children, and typically developing control adolescents. Post hoc analyses using Tukey’s Honestly Significant Difference (HSD) test were run for statistically significant results. Partial *η*^2^ was used to estimate effect size. Following this analysis, exploratory analysis into the correlations between age and social motivation within participant groups (i.e., typically developing children, children with ADHD, typically developing adolescents, adolescents with ADHD) were conducted using Pearson’s correlations.

To examine the relationship between social motivation and social anxiety symptoms in children and adolescents with ADHD, a linear regression was conducted with social anxiety symptoms as the independent variable (raw SCAS-P Social Phobia subscale scores) and social motivation as the dependent variable (raw SRS-2 Social Motivation subscale scores). Cohen’s *f* ^*2*^ was used to estimate effect size. Since there was no expected difference in social motivation between typically developing children and adolescents, only participants with ADHD were used in this analysis. The assumption of linearity was met, as determined through the inspection of plots (Field, 2009).

## Results

### Participant characteristics

Chi-square comparisons were run to assess whether there were any significant differences between groups regarding participant demographics. Groups did not differ in terms of gender, ADHD type, and medication status (*p* < .05). There were significant differences between participants with ADHD and typically developing participants for attentional (*p* < .001; Cramer’s V = .66) and social difficulties (*p* < .001; Cramer’s V = .28).

T-tests were run to assess whether there were any significant differences between age within child and adolescent participant groups. There were no significant differences in age between adolescents with and without ADHD (*p* > .05), however children with ADHD significantly differed in age from children without ADHD (*p* < .001, Cohen’s *d* = .86, constituting a large effect).

### Social motivation differences in children and adolescents with and without ADHD

Raw SRS-2 Social Motivation subscale scores for typically developing children (*M* = 7.49; *SD* = 4.84), children with ADHD (*M* = 8.85; *SD* = 6.28), typically developing adolescents (*M* = 8.89, *SD* = 5.68), and adolescents with ADHD (*M* = 13.10; *SD* = 5.76) were analysed with a one-way ANOVA. The result was statistically significant, *F*(3, 200) = 11.51, *p* < .001, η_p_^2^ = .15, indicating a large effect size.

**Figure 1.**
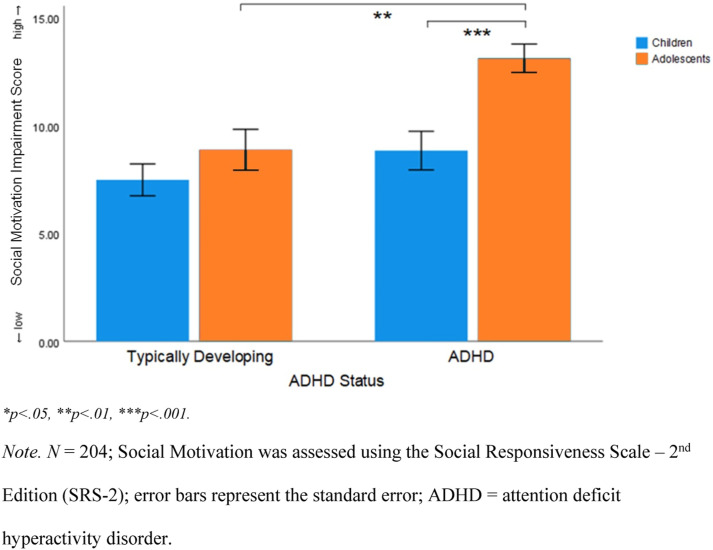
Mean Social Motivation Impairment Scores Between Children and Adolescents With and Without ADHD. *p < .05, **p < .01, ***p < .001. Note. *N* = 204; Social Motivation was assessed using the Social Responsiveness Scale – second Edition (SRS-2); error bars represent the standard error; ADHD: attention deficit hyperactivity disorder.

Tukey’s HSD post hoc comparisons revealed significant differences in social motivation impairment between adolescents with ADHD and typically developing adolescents; adolescents with ADHD had higher social motivation impairment scores than typically developing adolescents (*M*_Diff_ = 4.24, *p* = .002, 95% CI[1.26, 7.23]). A significant difference between adolescents with ADHD and children with ADHD was also found, whereby adolescents with ADHD had significantly higher social motivation impairment scores (*M*_Diff_ = 4.27, *p* < .001, 95% CI [1.58, 6.99]). A significant difference was also found between adolescents with ADHD and typically developing children, whereby adolescents with ADHD had significantly higher social motivation impairment scores (*M*_Diff_ = 5.64, *p* < .001, 95% CI [2.83, 8.46]). No significant differences in social motivation were found between children with ADHD and typically developing children, and typically developing children and typically developing adolescents. See Figure 1 for a visual representation of mean social motivation impairment comparisons between groups.

### Exploratory analysis into age-related differences in social motivation

Pearson’s correlations were run to assess whether there were significant within-group correlations between age and social motivation (SRS-2 Social Motivation subscale scores). There was a significant negative correlation present in the typically developing child group, and a significant positive correlation present in adolescents with ADHD. No other significant correlations were present. See Table 2 for correlation coefficients and significance.

**Table 2.** Correlation Coefficients (Pearson’s r) and Significance Values (p) for SRS-2 Social Motivation Subscale Scores and Age by Participant Group.

Participant group	Social motivation impairment	
	*r*	*p*
Children with ADHD	−.11	.462
Typically developing children	−.32	.035*
Adolescents with ADHD	.25	.028*
Typically developing adolescents	.19	.276

*p < .05, **p < .01, ***p < .001. Note. *N* = 204; social motivation impairment was measured by SRS-2 Social Motivation subscale scores, where higher scores indicate lower levels of social motivation; ADHD: attention deficit hyperactivity disorder.

### Social anxiety predicting social motivation

**Figure 2.**
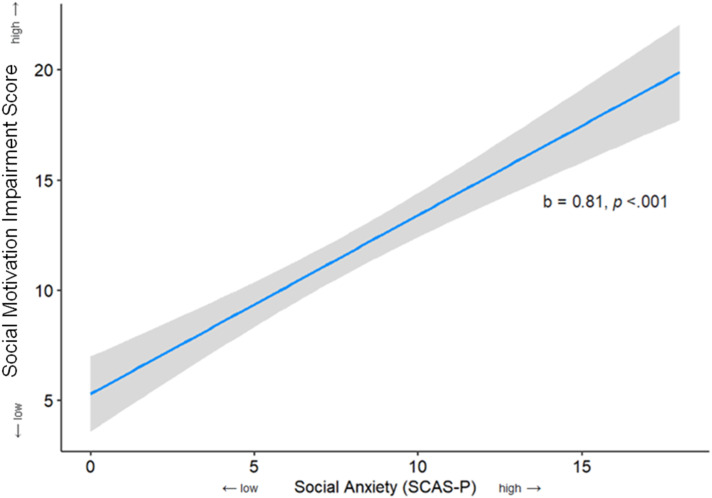
Relationship Between Social Anxiety and Social Motivation in Children and Adolescents with ADHD. Note. *N* = 204; social motivation impairment was measured by the SRS-2 Social Motivation subscale; higher scores on the *Y*-axis indicate higher levels of social motivation impairment; higher scores on the *X*-axis indicate higher levels of social anxiety; shaded region indicates the 95% confidence interval; the blue line indicates the linear relationship between social anxiety and social motivation; SRS-2: Social Responsiveness Scale – second Edition; SCAS-P: Spence Children’s Anxiety Scale – Parent.

A linear regression analysis was conducted to examine if social anxiety symptoms (SCAS-P Social Phobia subscale scores) could predict social motivation (SRS-2 Social Motivation subscale scores) in participants with ADHD. There was a statistically significant positive relationship between social anxiety symptoms and social motivation impairment. Each one-unit higher social anxiety score was associated with a .81 unit increase in social motivation impairment score, (95% CI [.62, 1.00], *p* < .001), which constituted a large effect ( *f* ^*2*^ = .58). The overall model explained 37% of the variance in social motivation (adjusted *R*^*2*^ = .36). See Figure 2 for a graph of this association.

## Discussion

The present study aimed to examine differences in the social motivation of children and adolescents with and without ADHD, and investigate the relationship between social motivation and social anxiety in children and adolescents with ADHD. The only significant differences found were that adolescents with ADHD had significantly lower parent-rated social motivation than typically developing control adolescents, and children with ADHD (a large effect). Additionally, as higher social anxiety symptoms significantly predicted lower social motivation in children and adolescents with ADHD (a large effect).

### Lowered social motivation in adolescents with ADHD

As hypothesised, adolescents with ADHD had significantly lower levels of social motivation than both their typically developing control counterparts, and children with ADHD. This is consistent with current preliminary literature investigating social motivation in children and adolescents with ADHD, which suggests that social motivation deficits in ADHD do not arise until adolescence (McKay et al., 2023; Smith & Langberg, 2018). Furthermore, this study found that typically developing children and adolescents did not significantly differ in their social motivation, whereas adolescents with ADHD had significantly lower levels of social motivation than children with ADHD. This suggests that the social motivation deficits in ADHD are not present from early childhood, and do not peak in adolescence as in typically developing populations (Ciranka & van den Bos, 2019).

Further supporting this finding, exploratory analyses into the correlations between age and social motivation impairment within participant groups yielded a significant positive correlation in adolescents with ADHD, suggesting that social motivation declines from ages 13 to 17. Additionally, no significant correlation was found between social motivation impairment and age in children with ADHD, contrasting the significant negative correlation (i.e., social motivation increases with age) observed in typically developing children. It is important to note that there was also no significant correlation present between age and social motivation in typically developing adolescents, contrasting the significant correlation expected if social motivation peaked in adolescence. Therefore, it is possible that both typically developing adolescents and adolescents with ADHD experienced lowered social motivation in the present study. This may be due to the present study involving samples of Australian respondents collected during the COVID-19 pandemic which was characterised by social distancing and discouraged social interactions, however further research into social motivation following COVID-19 is required to substantiate this claim. Taken together, consistent with previous suggestions (McKay et al., 2023; Smith & Langberg, 2018), the present study provides further evidence that social motivation appears to remain stable during childhood, and decline during adolescence for individuals with ADHD.

An explanation for why adolescents, but not children with ADHD may experience lower levels of social motivation than their typically developing peers surrounds their experiences of peer rejection. Children and adolescents with ADHD experience significantly greater instances of peer rejection as compared with their typically developing peers (Rokeach & Wiener, 2022). Whilst occasional rejection may temporarily increase social motivation, chronic rejection operates conversely (Vanhalst et al., 2018). Thus, by adolescence, children with ADHD may begin to anticipate rejection during peer interactions, reducing their motivation to pursue and engage in future social interactions (i.e., social motivation). This relationship between social motivation and peer rejection may be self-perpetuating; lowered social motivation reduces exposure to peer interactions in adolescents with ADHD, compounding existing social difficulties, increasing future incidences of peer rejection, and further lowering social motivation. However, more research into the predictors of social motivation in youth with ADHD across developmental periods is needed to substantiate this hypothesis.

### Higher social anxiety symptoms predict lower social motivation in ADHD

Low social motivation may be attributed to a lack of interest in social interactions and/or an avoidance of social interactions due to social anxiety. In the present study, higher social anxiety symptoms significantly predicted lower social motivation in children and adolescents with ADHD. This study was the first to investigate this relationship within children and adolescents with ADHD, however literature examining other neurodevelopmental disorders has indicated a similar relationship between social anxiety and social motivation (Cholemkery et al., 2014; South et al., 2017; Swain et al., 2015). A proposed explanation for this relationship surrounds the avoidance behaviours associated with social anxiety symptoms, whereby increased social anxiety reduces the tendency of individuals to seek out, foster, and enjoy social situations (i.e., social motivation; Bianchi et al., 2020; Bishop et al., 2019; Mikami, 2015). In the context of heightened social anxiety symptoms in children and adolescents with ADHD, repeated incidences of peer rejection may act to increase social anxiety symptoms and their associated avoidance behaviours; in turn these exacerbate current social difficulties, and decrease social motivation. This relationship between social anxiety and social motivation may be bidirectional, as recent literature has indicated that the avoidance of anxiety-inducing situations (i.e., social situations) is associated with increased social anxiety symptoms (Papachristou et al., 2018). Overall, the findings from this study provide novel evidence to suggest the presence of a negative relationship between social anxiety symptoms and social motivation in children and adolescents with ADHD.

The results from the present study have implications for understanding the potential barriers to improving social skills in adolescents with ADHD. The atypically low social motivation observed in adolescents with ADHD may act to reduce the engagement in, and therefore the observed benefit from, social interventions in this population. Moreover, as higher social anxiety symptoms significantly predicted lower social motivation in children and adolescents with ADHD, social anxiety symptoms may act as a further barrier to social intervention efficacy. Therefore, adolescents with ADHD who experience social anxiety symptoms may be even less likely to engage in and perform learnt skills outside of social intervention sessions, reflecting the current inefficacy of interventions within this population (Bishop et al., 2019; Mikami, 2015). Consequently, when assessing the efficacy of social interventions for adolescents with ADHD, researchers may consider administering a measure of social motivation and social anxiety to gauge whether intervention inefficacy is due to issues in program design, or potential barriers to treatment engagement and social performance.

The current study also has clinical implications. Currently, children and adolescents with ADHD and social anxiety symptoms experience little benefits from available social interventions (Bishop et al., 2019; Mikami, 2015). Screening for and addressing social anxiety in adolescents with ADHD may improve social motivation within this population, given the likely bi-directional relationship between these factors (McKay et al., 2023). Providing strategies to all individuals with ADHD (including those without social anxiety) to improve management and reduce the occurrence of social anxiety symptoms may also act as a protective factor for social motivation. Furthermore, children with ADHD appeared to experience similarly high levels of social motivation to their typically developing peers, and had significantly higher levels of social motivation than adolescents with ADHD. Consistent with past research (Devine & Apperly, 2022; Leary & Allen, 2011), this suggests that social interventions applied during childhood may therefore yield better outcomes due to the enhanced engagement and social performance accompanying high social motivation. Not only would this reduce the burden on the parent and child, as they avoid engaging in multiple ineffective interventions throughout adolescence, but it may also protect against future incidences of peer rejection, hence reducing the potential for social motivation to decline later in life (Vanhalst et al., 2018).

### Limitations and future directions

Despite its many strengths (i.e., representative sample, novelty, large sample size) this study was not without limitations. First, the use of a cross-sectional design and lack of data for 9–12-year-old children impeded conclusions surrounding the trajectory of social motivation, and the directionality of identified relationships. However, the negative impacts of COVID-19 lockdowns on social functioning during the present study may have confounded longitudinal results (Lestari et al., 2022). Additionally, although there were no significant differences found in the social motivation of children with and without ADHD, these two groups significantly differed in age, reducing group comparability. Whilst typically considered a valid measure of anxiety symptoms in children, the use of only parent report measures of social anxiety is a limitation, as the use of multiple reporters is preferred where possible. Finally, demographic data surrounding ethnicity and socioeconomic status were not collected in the present study. Longitudinal research using age-matched samples and controlling for socioeconomic status and ethnicity is therefore required to corroborate the present study’s preliminary findings.

Given that high social motivation has been associated with increased intervention engagement and skills application (Devine & Apperly, 2022), it is important for future research to further investigate the relationship between social motivation and social outcomes in children and adolescents with ADHD. Additionally, to increase understandings of why social motivation deficits exist in adolescents, but not children with ADHD, prospective research should investigate the role of peer rejection.

## Conclusion

In summary, this was the first known study to compare levels of social motivation between children and adolescents with and without ADHD, and investigate the role of social anxiety as a predictor of social motivation in ADHD. Adolescents with ADHD had significantly lower levels of social motivation than both children with ADHD, and typically developing adolescents. Moreover, higher levels of social anxiety symptoms predicted lower levels of social motivation in children and adolescents with ADHD. Social motivation and social anxiety may therefore act as potential barriers to social intervention efficacy for adolescents with ADHD, indicating that it may be beneficial for clinicians and researchers to consider these factors prior to administering social interventions.
